# (*E*)-4-Methyl-*N*′-(3-nitro­benzyl­idene)benzohydrazide

**DOI:** 10.1107/S1600536812019198

**Published:** 2012-05-05

**Authors:** Hua-Nan Hu, Shi-Yong Liu

**Affiliations:** a College of Chemistry & Pharmacy, Taizhou University, Taizhou Zhejiang 317000, People’s Republic of China

## Abstract

In the title compound, C_15_H_13_N_3_O_3_, the dihedral angle between the benzene rings is 1.01 (3)° and that between the nitro group and its attached ring is 5.99 (15)°. In the crystal, mol­ecules are linked by N—H⋯O hydrogen bonds generating *C*(4) chains along [010].

## Related literature

For related structures, see: Liu & You (2010 ▶); Liu & Wang (2010 ▶); Xu *et al.* (2009 ▶); Shafiq *et al.* (2009 ▶).
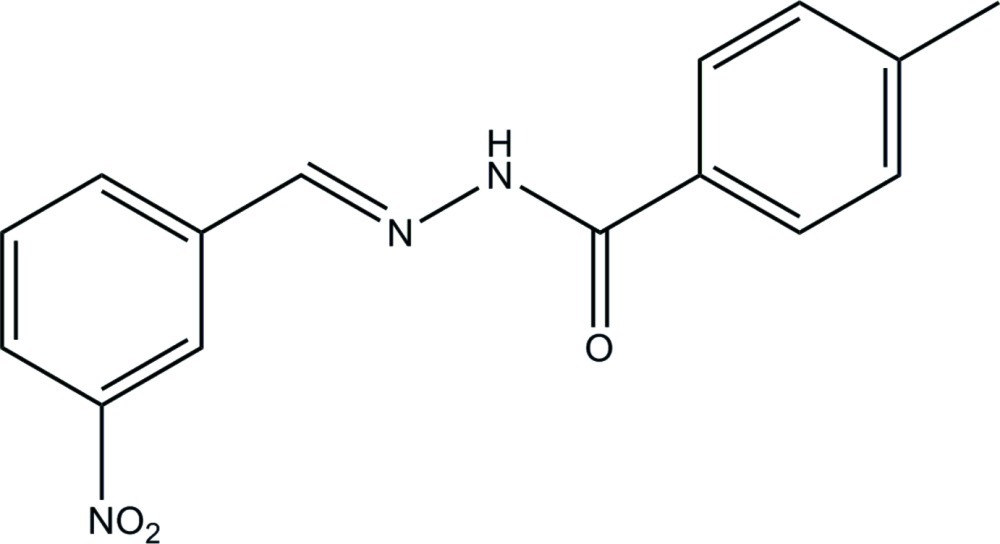


## Experimental

### 

#### Crystal data

C_15_H_13_N_3_O_3_
*M* *_r_* = 283.28
Orthorhombic, 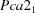

*a* = 32.657 (3) Å
*b* = 4.7861 (15) Å
*c* = 8.7596 (12) Å
*V* = 1369.1 (5) Å^3^
*Z* = 4
Mo *K*α radiation
μ = 0.10 mm^−1^
*T* = 298 K
0.20 × 0.20 × 0.17 mm

#### Data collection

Bruker SMART CCD diffractometer
Absorption correction: multi-scan (*SADABS*; Bruker, 2001 ▶) *T*_min_ = 0.981, *T*_max_ = 0.984
9086 measured reflections
2539 independent reflections
2036 reflections with *I* > 2σ(*I*)
*R*_int_ = 0.045

#### Refinement

*R*[*F* ^2^ > 2σ(*F* ^2^)] = 0.049
*wR*(*F* ^2^) = 0.103
*S* = 1.04
2539 reflections
195 parameters
2 restraints
H atoms treated by a mixture of independent and constrained refinement
Δρ_max_ = 0.13 e Å^−3^
Δρ_min_ = −0.17 e Å^−3^

### 

Data collection: *SMART* (Bruker, 2007 ▶); cell refinement: *SAINT* (Bruker, 2007 ▶); data reduction: *SAINT*; program(s) used to solve structure: *SHELXTL* (Sheldrick, 2008 ▶); program(s) used to refine structure: *SHELXTL*; molecular graphics: *SHELXTL*; software used to prepare material for publication: *SHELXTL*.

## Supplementary Material

Crystal structure: contains datablock(s) global, I. DOI: 10.1107/S1600536812019198/hb6769sup1.cif

Structure factors: contains datablock(s) I. DOI: 10.1107/S1600536812019198/hb6769Isup2.hkl

Supplementary material file. DOI: 10.1107/S1600536812019198/hb6769Isup3.cml

Additional supplementary materials:  crystallographic information; 3D view; checkCIF report

## Figures and Tables

**Table 1:** Hydrogen-bond geometry (Å, °)

*D*—H⋯*A*	*D*—H	H⋯*A*	*D*⋯*A*	*D*—H⋯*A*
N3—H3⋯O3^i^	0.89 (1)	2.13 (2)	2.909 (3)	146 (2)

Symmetry code: (i) 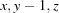.

**Fig. 1.**
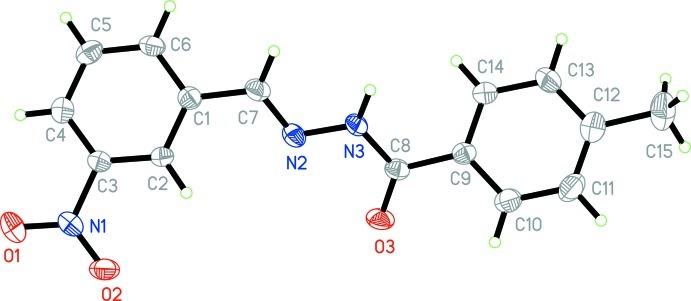
The molecular structure of the title compound. Displacement ellipsoids are drawn at the 30% probability level.

**Fig. 2.**
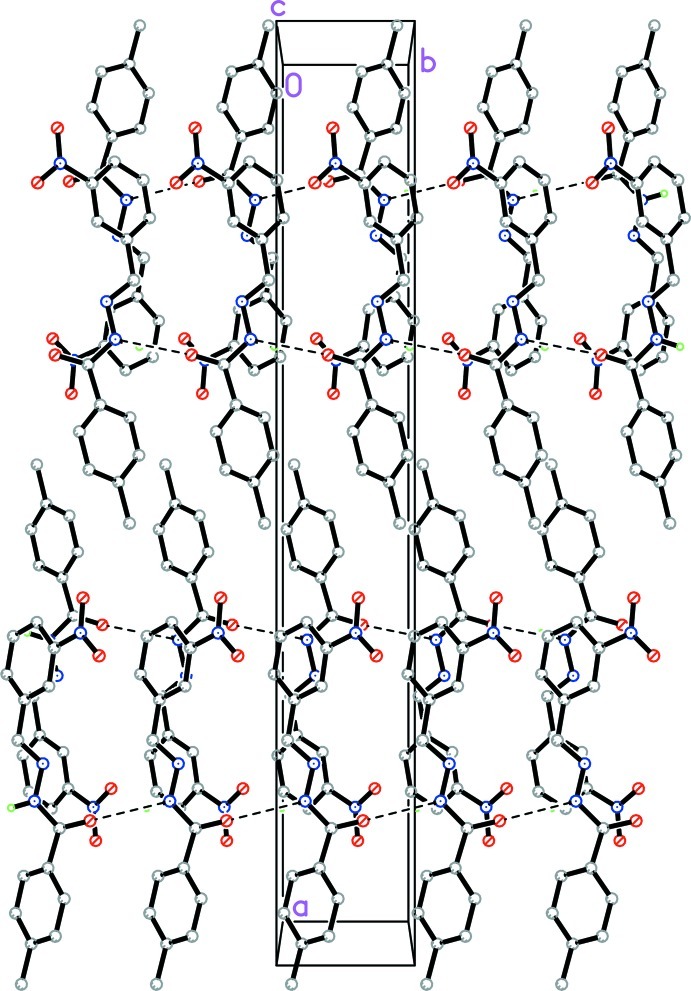
The molecular packing of the title compound, viewed along the c axis. Hydrogen bonds are shown as dashed lines. Hydrogen atoms not involved in hydrogen bonding have been omitted.

## supplementary crystallographic information

### Comment

As a continuation of our work on similar compounds (Liu & You, 2010; Liu & Wang, 2010), we report herein the crystal structure of the title compound a new hydrazone.

The molecular structure of the title compound is shown in Fig. 1. The two benzene ring system are inclined at a dihedral angle of 0.6 (3)°. All the bond lengths are comparable to those observed in related structures (Xu *et al.*, 2009; Shafiq *et al.*, 2009) and those we reported previously.

In the crystal structure, molecules are linked through N–H···O hydrogen bonds, to form one-dimensional chains running along the *b* axis (Fig. 2 and Table 1).

### Experimental

The title compound was prepared by the condensation reaction of 3-nitrobenzaldehyde (0.05 mol, 7.6 g) and 4-methylbenzohydrazide (0.05 mol, 7.5 g) in anhydrous methanol (100 ml) at ambient temperature. Yellow block-shaped single crystals were obtained by slow evaporation of the solution for several days.

### Refinement

Anomalous dispersion was negligible and the absolute structure could not be determined in the present experiment. H3 was located from a difference Fourier map and refined isotropically, with the N–H distance restrained to 0.90 (1) Å. The remaining H atoms were positioned geometrically and constrained to ride on their parent atoms, with C–H distances of 0.93–0.96 Å, and with *U*_iso_(H) = 1.2*U*_eq_(C) and 1.5*U*_eq_(C15).

### Crystal data

**Table:**

C_15_H_13_N_3_O_3_	*D*_x_ = 1.374 Mg m^−^^3^
*M**_r_* = 283.28	Mo *K*α radiation, λ = 0.71073 Å
Orthorhombic, *P**c**a*2_1_	Cell parameters from 1981 reflections
*a* = 32.657 (3) Å	θ = 2.5–24.3°
*b* = 4.7861 (15) Å	µ = 0.10 mm^−^^1^
*c* = 8.7596 (12) Å	*T* = 298 K
*V* = 1369.1 (5) Å^3^	Block, yellow
*Z* = 4	0.20 × 0.20 × 0.17 mm
*F*(000) = 592	

### Data collection

**Table:**

Bruker SMART CCD diffractometer	2539 independent reflections
Radiation source: fine-focus sealed tube	2036 reflections with *I* > 2σ(*I*)
Graphite monochromator	*R*_int_ = 0.045
ω scans	θ_max_ = 25.5°, θ_min_ = 2.5°
Absorption correction: multi-scan (*SADABS*; Bruker, 2001)	*h* = −39→37
*T*_min_ = 0.981, *T*_max_ = 0.984	*k* = −5→5
9086 measured reflections	*l* = −10→10

### Refinement

**Table:**

Refinement on *F*^2^	Primary atom site location: structure-invariant direct methods
Least-squares matrix: full	Secondary atom site location: difference Fourier map
*R*[*F*^2^ > 2σ(*F*^2^)] = 0.049	Hydrogen site location: inferred from neighbouring sites
*wR*(*F*^2^) = 0.103	H atoms treated by a mixture of independent and constrained refinement
*S* = 1.04	*w* = 1/[σ^2^(*F*_o_^2^) + (0.0473*P*)^2^ + 0.1113*P*] where *P* = (*F*_o_^2^ + 2*F*_c_^2^)/3
2539 reflections	(Δ/σ)_max_ < 0.001
195 parameters	Δρ_max_ = 0.13 e Å^−^^3^
2 restraints	Δρ_min_ = −0.17 e Å^−^^3^

### Special details

**Table:**

Geometry. All esds (except the esd in the dihedral angle between two l.s. planes) are estimated using the full covariance matrix. The cell esds are taken into account individually in the estimation of esds in distances, angles and torsion angles; correlations between esds in cell parameters are only used when they are defined by crystal symmetry. An approximate (isotropic) treatment of cell esds is used for estimating esds involving l.s. planes.
Refinement. Refinement of F^2^ against ALL reflections. The weighted R-factor wR and goodness of fit S are based on F^2^, conventional R-factors R are based on F, with F set to zero for negative F^2^. The threshold expression of F^2^ > 2sigma(F^2^) is used only for calculating R-factors(gt) etc. and is not relevant to the choice of reflections for refinement. R-factors based on F^2^ are statistically about twice as large as those based on F, and R- factors based on ALL data will be even larger.

### Fractional atomic coordinates and isotropic or equivalent isotropic displacement parameters (Å^2^)

**Table:**

	*x*	*y*	*z*	*U*_iso_*/*U*_eq_	
N1	0.85121 (8)	0.5734 (5)	0.4452 (2)	0.0467 (6)	
N2	0.70734 (7)	0.2646 (4)	0.2869 (3)	0.0444 (5)	
N3	0.66666 (7)	0.2015 (4)	0.2812 (3)	0.0455 (5)	
O1	0.88853 (7)	0.5929 (5)	0.4514 (3)	0.0749 (7)	
O2	0.82822 (7)	0.7214 (4)	0.5169 (2)	0.0597 (6)	
O3	0.64841 (6)	0.6273 (4)	0.3664 (2)	0.0553 (5)	
C1	0.77578 (8)	0.1201 (5)	0.2510 (3)	0.0413 (6)	
C2	0.79253 (8)	0.3213 (5)	0.3444 (3)	0.0377 (6)	
H2	0.7758	0.4289	0.4072	0.045*	
C3	0.83377 (8)	0.3605 (5)	0.3438 (3)	0.0382 (6)	
C4	0.85976 (8)	0.2118 (6)	0.2508 (3)	0.0488 (7)	
H4	0.8878	0.2448	0.2521	0.059*	
C5	0.84325 (9)	0.0139 (6)	0.1563 (3)	0.0527 (7)	
H5	0.8601	−0.0881	0.0914	0.063*	
C6	0.80177 (8)	−0.0341 (5)	0.1572 (3)	0.0457 (7)	
H6	0.7909	−0.1717	0.0942	0.055*	
C7	0.73153 (8)	0.0702 (6)	0.2463 (3)	0.0479 (7)	
H7	0.7213	−0.1012	0.2140	0.057*	
C8	0.63869 (8)	0.3942 (5)	0.3216 (3)	0.0418 (6)	
C9	0.59528 (8)	0.3070 (5)	0.3091 (3)	0.0405 (6)	
C10	0.56597 (9)	0.4440 (6)	0.3943 (4)	0.0591 (8)	
H10	0.5739	0.5841	0.4617	0.071*	
C11	0.52522 (10)	0.3763 (7)	0.3809 (4)	0.0677 (9)	
H11	0.5062	0.4680	0.4418	0.081*	
C12	0.51204 (9)	0.1772 (7)	0.2801 (4)	0.0600 (8)	
C13	0.54139 (9)	0.0401 (7)	0.1948 (3)	0.0622 (9)	
H13	0.5333	−0.0988	0.1269	0.075*	
C14	0.58242 (9)	0.1047 (6)	0.2083 (3)	0.0533 (8)	
H14	0.6015	0.0107	0.1488	0.064*	
C15	0.46734 (9)	0.0998 (9)	0.2613 (5)	0.0889 (12)	
H15A	0.4505	0.2489	0.2990	0.133*	
H15B	0.4617	−0.0678	0.3177	0.133*	
H15C	0.4615	0.0692	0.1551	0.133*	
H3	0.6588 (7)	0.025 (3)	0.266 (3)	0.050 (8)*	

### Atomic displacement parameters (Å^2^)

**Table:**

	*U*^11^	*U*^22^	*U*^33^	*U*^12^	*U*^13^	*U*^23^
N1	0.0515 (16)	0.0457 (13)	0.0428 (13)	−0.0090 (12)	−0.0092 (12)	0.0008 (11)
N2	0.0438 (14)	0.0381 (12)	0.0512 (12)	−0.0091 (10)	−0.0044 (11)	−0.0003 (11)
N3	0.0398 (13)	0.0312 (12)	0.0655 (15)	−0.0094 (10)	0.0000 (11)	−0.0075 (12)
O1	0.0505 (14)	0.0829 (16)	0.0915 (17)	−0.0158 (12)	−0.0164 (12)	−0.0160 (13)
O2	0.0715 (14)	0.0567 (13)	0.0507 (12)	−0.0013 (12)	0.0013 (10)	−0.0194 (11)
O3	0.0679 (13)	0.0328 (10)	0.0651 (12)	−0.0097 (9)	0.0031 (11)	−0.0116 (9)
C1	0.0447 (16)	0.0338 (14)	0.0456 (15)	0.0006 (12)	−0.0042 (13)	0.0025 (12)
C2	0.0428 (16)	0.0323 (12)	0.0379 (14)	0.0017 (11)	0.0006 (11)	−0.0043 (11)
C3	0.0446 (15)	0.0355 (13)	0.0343 (14)	−0.0024 (12)	−0.0025 (12)	−0.0003 (11)
C4	0.0428 (16)	0.0509 (17)	0.0526 (17)	0.0012 (13)	−0.0004 (14)	0.0031 (15)
C5	0.0582 (19)	0.0543 (18)	0.0456 (15)	0.0114 (14)	0.0079 (15)	−0.0043 (15)
C6	0.0583 (18)	0.0373 (15)	0.0415 (14)	0.0008 (13)	−0.0084 (14)	−0.0067 (13)
C7	0.0512 (18)	0.0368 (15)	0.0555 (17)	−0.0060 (13)	−0.0034 (14)	−0.0054 (13)
C8	0.0505 (16)	0.0337 (14)	0.0412 (15)	−0.0047 (12)	0.0005 (12)	−0.0008 (12)
C9	0.0473 (16)	0.0310 (13)	0.0433 (15)	0.0013 (11)	0.0024 (12)	0.0023 (11)
C10	0.062 (2)	0.0535 (17)	0.0621 (19)	−0.0044 (15)	0.0135 (16)	−0.0127 (16)
C11	0.059 (2)	0.065 (2)	0.078 (2)	0.0081 (17)	0.0222 (18)	−0.007 (2)
C12	0.0511 (19)	0.068 (2)	0.0614 (19)	0.0021 (15)	0.0041 (16)	0.0096 (18)
C13	0.055 (2)	0.071 (2)	0.0607 (19)	−0.0138 (16)	−0.0062 (15)	−0.0175 (16)
C14	0.0497 (19)	0.0525 (18)	0.0577 (18)	−0.0012 (14)	0.0035 (13)	−0.0163 (14)
C15	0.048 (2)	0.127 (3)	0.091 (3)	−0.002 (2)	0.000 (2)	0.008 (3)

### Geometric parameters (Å, º)

**Table:**

N1—O2	1.208 (3)	C6—H6	0.9300
N1—O1	1.224 (3)	C7—H7	0.9300
N1—C3	1.467 (3)	C8—C9	1.482 (3)
N2—C7	1.272 (3)	C9—C14	1.376 (3)
N2—N3	1.364 (3)	C9—C10	1.379 (4)
N3—C8	1.345 (3)	C10—C11	1.375 (4)
N3—H3	0.891 (10)	C10—H10	0.9300
O3—C8	1.225 (3)	C11—C12	1.369 (5)
C1—C2	1.377 (3)	C11—H11	0.9300
C1—C6	1.393 (4)	C12—C13	1.381 (4)
C1—C7	1.465 (3)	C12—C15	1.515 (4)
C2—C3	1.360 (3)	C13—C14	1.380 (4)
C2—H2	0.9300	C13—H13	0.9300
C3—C4	1.375 (4)	C14—H14	0.9300
C4—C5	1.369 (4)	C15—H15A	0.9600
C4—H4	0.9300	C15—H15B	0.9600
C5—C6	1.374 (4)	C15—H15C	0.9600
C5—H5	0.9300		
			
O2—N1—O1	123.4 (2)	O3—C8—N3	122.2 (2)
O2—N1—C3	118.7 (2)	O3—C8—C9	121.9 (2)
O1—N1—C3	117.8 (2)	N3—C8—C9	115.9 (2)
C7—N2—N3	115.6 (2)	C14—C9—C10	118.0 (3)
C8—N3—N2	120.0 (2)	C14—C9—C8	122.5 (2)
C8—N3—H3	119.5 (16)	C10—C9—C8	119.4 (2)
N2—N3—H3	119.7 (16)	C11—C10—C9	120.9 (3)
C2—C1—C6	118.6 (2)	C11—C10—H10	119.5
C2—C1—C7	121.5 (2)	C9—C10—H10	119.5
C6—C1—C7	119.9 (2)	C12—C11—C10	121.6 (3)
C3—C2—C1	119.2 (2)	C12—C11—H11	119.2
C3—C2—H2	120.4	C10—C11—H11	119.2
C1—C2—H2	120.4	C11—C12—C13	117.5 (3)
C2—C3—C4	122.8 (2)	C11—C12—C15	122.9 (3)
C2—C3—N1	118.6 (2)	C13—C12—C15	119.6 (3)
C4—C3—N1	118.6 (2)	C14—C13—C12	121.4 (3)
C5—C4—C3	118.3 (3)	C14—C13—H13	119.3
C5—C4—H4	120.9	C12—C13—H13	119.3
C3—C4—H4	120.9	C9—C14—C13	120.6 (3)
C4—C5—C6	120.0 (3)	C9—C14—H14	119.7
C4—C5—H5	120.0	C13—C14—H14	119.7
C6—C5—H5	120.0	C12—C15—H15A	109.5
C5—C6—C1	121.0 (2)	C12—C15—H15B	109.5
C5—C6—H6	119.5	H15A—C15—H15B	109.5
C1—C6—H6	119.5	C12—C15—H15C	109.5
N2—C7—C1	119.0 (2)	H15A—C15—H15C	109.5
N2—C7—H7	120.5	H15B—C15—H15C	109.5
C1—C7—H7	120.5		

### Hydrogen-bond geometry (Å, º)

**Table:**

*D*—H···*A*	*D*—H	H···*A*	*D*···*A*	*D*—H···*A*
N3—H3···O3^i^	0.89 (1)	2.13 (2)	2.909 (3)	146 (2)

Symmetry code: (i) *x*, *y*−1, *z*.
